# SERCA2a dysfunction in the pathophysiology of heart failure with preserved ejection fraction: a direct role is yet to be established

**DOI:** 10.1007/s10741-025-10487-1

**Published:** 2025-01-23

**Authors:** Adam Kia Shooshtarian, Kevin O’Gallagher, Ajay M. Shah, Min Zhang

**Affiliations:** School of Cardiovascular and Metabolic Medicine & Sciences, King’s College London British Heart Foundation Centre of Research Excellence, London, UK

**Keywords:** Heart failure, Ejection fraction, SERCA2a, Diastolic dysfunction, Calcium regulation

## Abstract

With rising incidence, mortality and limited therapeutic options, heart failure with preserved ejection fraction (HFpEF) remains one of the most important topics in cardiovascular medicine today. Characterised by left ventricular diastolic dysfunction partially due to impaired Ca^2+^ homeostasis, one ion channel in particular, SarcoEndoplasmic Reticulum Ca^2+^-ATPase (SERCA2a), may play a significant role in its pathophysiology. A better understanding of the complex mechanisms interplaying to contribute to SERCA2a dysfunction will help develop treatments targeting it and thus address the growing clinical challenge HFpEF poses. This review examines the conflicting evidence present for changes in SERCA2a expression and activity in HFpEF, explores potential underlying mechanisms, and finally evaluates the drug and gene therapy trials targeting SERCA2a in heart failure. Recent positive results from trials involving widely used anti-diabetic agents such as sodium-glucose co-transporter protein 2 inhibitors (SGLT2i) and glucagon-like peptide-1 (GLP-1) agonists offer advancement in HFpEF management. The potential interplay between these agents and SERCA2a regulation presents a novel angle that could open new avenues for modulating diastolic function; however, the mechanistic research in this emerging field is limited. Overall, the direct role of SERCA2a dysfunction in HFpEF remains undetermined, highlighting the need for well-designed pre-clinical studies and robust clinical trials.

## Introduction

The global incidence of heart failure (HF) is increasing, with approximately 6.7 million Americans aged 20 years or above suffering from the disease [1]. There are three proposed subtypes of HF, with patients being classified in accordance with echocardiographic left ventricular ejection fraction (LVEF) measurements. Those with heart failure and reduced ejection fraction (HFrEF) exhibit LVEF levels ≤ 40%, whilst those with HFpEF maintain a LVEF of ≥ 50%. An intermediate group, heart failure with mildly reduced ejection fraction (HFmrEF), encompasses patients with a LVEF of 41–49%, as per the 2023 focused update of the 2021 European society of cardiology (ESC) guidelines [2]. Approximately 50% of HF patients are now classified as having HFpEF [3]. Whilst there are established pharmacological treatments for HFrEF targeting the neurohormonal activations that occur in the disease [4], effective treatment of HFpEF remains challenging due to its phenotypic heterogeneity [5] and the lack of an accepted common pathomechanism, ultimately resulting in a more limited therapeutic arsenal [6]. Moreover, the treatments currently available for HFpEF including sodium-glucose co-transporter 2 inhibitors (SGLT2i) and glucagon-like peptide 1 (GLP-1) agonists were not originally developed as part of the treatment for clinical syndrome, but rather for type 2 diabetes mellitus (T2DM) and obesity [7]. It was only recently discovered that their therapeutic efficacy extends beyond their initial indications, including positive benefits in HFpEF patients. Furthermore, a recent meta-analysis concluded that, despite the ability of drugs such as SGLT2i to reduce hospitalisation in HFpEF patients; as of yet, no treatment has been effective in reducing mortality [8].

Contraction and relaxation of cardiac myocytes are centrally modulated by the intracellular [Ca^2+^] [9], which, in turn, is regulated by movement of Ca^2+^ in and out of the sarcoplasmic reticulum (SR). A key protein involved in this movement is the SarcoEndoplasmic Reticulum Calcium ATPase (SERCA2a) pump found on the SR of cardiac myocytes [10]. SERCA2a dysfunction has long been implicated in the pathogenesis of HFrEF [11] however, there has been limited exploration of its specific role in HFpEF, leaving the precise mechanisms by which SERCA2a may (or may not) contribute to the clinical syndrome less well defined. This topic review aims to examine the past and current evidence regarding the changes and putative roles of SERCA2a in HFpEF, along with the potential underlying mechanisms. The discussion will extend to the drug and gene therapies targeting SERCA2a in HFpEF (and HFrEF), as well as recent developments in the treatment of HFpEF and how these may relate to SERCA2a.

## HFpEF: a clinical conundrum

First identified by Luchi et al. in 1982 [12], HFpEF can be defined as a complex syndrome in which the heart fails to maintain a cardiac output (CO) satisfactory to meet the metabolic requirements of the body tissue, with an EF ≥ 50% accompanied by clinical signs of heart failure [13]. Historically, HFrEF and HFpEF have been associated with systolic and diastolic dysfunction, respectively. There is, however, increasing evidence to suggest that HFpEF is more than just diastolic dysfunction and a degree of systolic dysfunction does occur too at rest, which exacerbates with exercise [14]. In addition, many HFrEF patients also exhibit diastolic dysfunction as well [15]. Whilst the exact aetiology is unclear, a range of risk factors have been implicated in the pathogenesis of the syndrome [16], including diabetes, obesity, hypertension, ageing, coronary artery disease and pulmonary hypertension [17]. It is important to clarify that this review excludes HFpEF attributed to secondary causes such as pulmonary emboli, pericardial disease or cardiac infiltration [18]. These conditions can mimic HFpEF but involve distinct pathophysiological mechanisms and therefore require different therapeutic approaches.

The diagnosis of HFpEF remains a challenge for clinicians and the condition is often under-diagnosed [19]. Current ESC criteria state that a diagnosis of HFpEF can be made in patients with signs and/or symptoms of HF, a LVEF ≥ 50%, and evidence of structural or functional cardiac abnormalities that are consistent with LV diastolic dysfunction and raised LV filling pressure/natriuretic peptide levels [2]. The ESC criteria remain broad, suggesting that the greater number of abnormalities present, the higher the likelihood of HFpEF [2]. This underscores the fact that there is still uncertainty surrounding the precise definition of HFpEF and that, beyond its systemic nature, there continues to be a lack of a comprehensive understanding of its underlying pathophysiology. A retrospective cohort study from our institution explored the application of natural language processing (NLP) to improve the diagnosis of HFpEF [20]. Mortality was reduced in patients with formally diagnosed HFpEF, highlighting both the importance in tackling the under-diagnosis of HFpEF and the need for physician review in these patients.

Techniques such as phenomapping offer a potential means to identify discrete sub-groups within the heterogeneous HFpEF population. Recent studies utilising the electronic patient record, machine learning or gene expression signatures reaffirm the long-standing notion that HFpEF patients comprise a wide phenotypic spectrum [21–23]. Notably, one study [21] proposed five sub-phenotypes: (1) natriuretic peptide (NP) deficiency, (2) cardiometabolic, (3) chronic cardiovascular risk and high filling pressures resulting in pulmonary hypertension, (4) atrial myopathy and (5) natural progression of (4), i.e., worsening mitral regurgitation. Although these studies aim to aid the diagnosis and classification of HFpEF, they further add to its complexity. The presence of distinct underlying phenotypes within the HFpEF population complicates matters further and may be responsible for the challenges in diagnosis, as well as elucidating the role SERCA2a may or may not play.

## Cardiac muscle contraction and relaxation—what role does SERCA2a play?

Cardiac diastole consists of two key phases: rapid ventricular filling (linked to isovolumic relaxation) and then late filling (linked to atrial contraction) [24]. The chief mechanism by which cardiac myocyte relaxation occurs is through ATP-driven uptake of calcium by the sarcoplasmic reticulum (SR) via SERCA2a and the dissociation of calcium from myofilaments. A study directly measuring the Ca^2+^ transport balance among various Ca^2+^ handling proteins found that in non-failing human cardiomyocytes, SERCA2a is responsible for 77% of the decline in intracellular calcium [Ca^2+^]_i_, whilst the Na^+^/Ca^2+^ exchanger (NCX) on the cell surface membrane accounts for the remaining 23% [25]. In HFrEF, these contributions shift to 64% for SERCA2a and 36% for NCX, indicating a 57% increase in NCX’s relative contribution, primarily due to reduced intrinsic function of SERCA2a. The mitochondrial uniporter and sarcolemmal Ca^2+^ ATPase also contribute, however to much lesser extent [26]. Regulation of SERCA2a occurs via the accessory protein, phospholamban (PLN) in the ventricles and by sarcolipin in the atria [27]. In a dephosphorylated state, specific residues in the transmembrane domain of PLN physically interact with SERCA2a, altering its tertiary structure, thereby exerting an inhibitory effect on SERCA2a through reducing its affinity for Ca^2+^ [28]. Binding of catecholamines, such as adrenaline and noradrenaline, to β−1 and β−2 adrenergic receptors (both G protein receptors coupled to Gs) results in formation of 3′,5′-cyclic adenosine monophosphate (cAMP) from ATP via adenylyl cyclase [29]. The increase in cAMP levels activates cAMP-dependent protein kinase A (PKA), which phosphorylates PLN at its Ser-16 residue, or calmodulin-dependent protein kinase (CAMKII), which phosphorylates PLN at the Thr-17 residue [30]. Some studies have reported that the former serves as a prerequisite for CaMKII-mediated phosphorylation of Thr-17 [31], whilst others claim these may both be independent processes [32]. Phosphorylation at these sites terminates PLN’s inhibitory effect by increasing the affinity of SERCA2a for Ca^2+^. This mechanism allows SERCA2a to contribute to the systolic Ca^2+^ transient (CaT) that allows cardiac contractility [33]. Secondary messenger signalling by cAMP is under precise control by A-kinase anchoring proteins (AKAPs), which anchor PKA near the PLN-SERCA2a complex within the SR and mediate phosphorylation of PLN at its Ser-16 residue, thus permitting re-uptake of Ca^2+^ [34]. AKAP-organised cAMP microdomains underwent remodelling in a mouse model of HFpEF [35], denoted by an increase in cAMP levels originating from β2-AR stimulation, leading to increased PLN phosphorylation and consequent increased influx of Ca^2+^ into the SR. This may have been due to downregulation of phosphodiesterase 4D/4B (PDE4B/4D), which reduces cAMP breakdown. This was accompanied by a reduction in cAMP in the PLN/SERCA2a microdomain that was derived from β1-AR desensitization. Whilst the phosphorylation state of PLN is a critical regulator of SERCA2a activity, other factors are also important in influencing SERCA2a activity such as the concentration of the cytoplasmic [Ca^2+^] and the SR Ca^2+^ content [36].

## Mechanisms of SERCA2a dysfunction

**Fig. 1:**
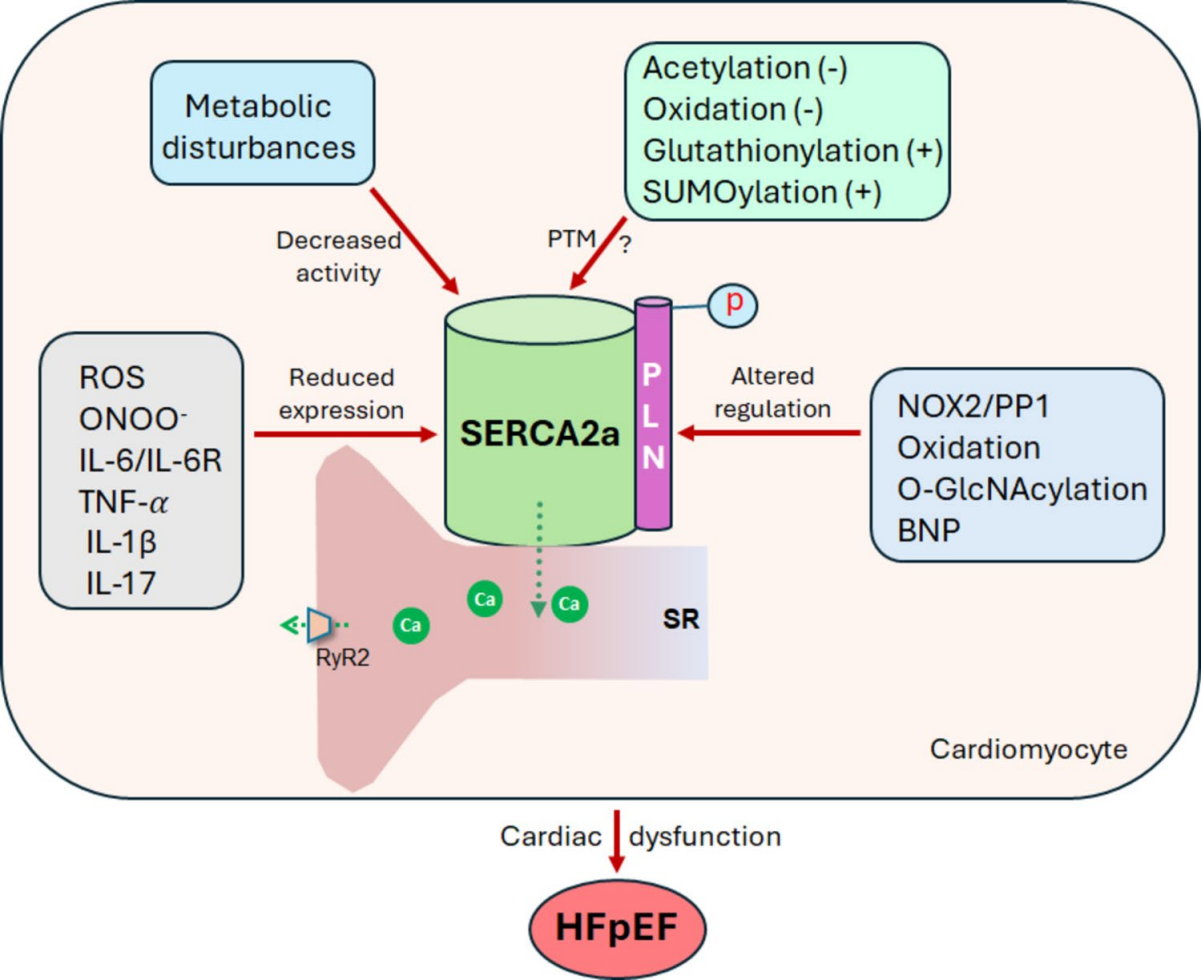
Potential mechanisms underlying SERCA2a dysfunction in HFpEF. SERCA2a expression and activity in the sarcoplasmic reticulum (SR) can be affected by various complex processes. Excessive reactive oxygen species (ROS) and oxidative stress react with nitric oxide (NO) to generate peroxynitrite (ONOO^−^), which increases interleukin-6 receptor (IL-6R) expression, inhibiting transcription of the gene encoding SERCA2a: *atp2a2*. Inflammation, predominantly through TNF-α, IL-1β and IL-17, suppresses SERCA2a gene expression. Phospholamban (PLN), which is the key regulator of SERCA2a activity, can be modulated by the increased NADPH oxidase 2 (NOX2) activation, elevated levels of B-type natriuretic peptide (BNP), and oxidation or O-GlcNAcylation of PLN. Insufficient ATP generation due to metabolic disturbances can reduce SERCA2a activity. Moreover, although SERCA2a activity can be modulated by post-translational modifications (PTM) such as acetylation and oxidative (decrease SERCA2a activity), Glutathionylation and SUMOylation (increase SERCA2a activity), the exact changes of PTM of SERCA2a in HFpEF warrant further investigation

It should be noted that to this date both the exact mechanisms responsible for HFpEF and SERCA2a dysfunction in HFpEF, remain not fully understood. Figure 1 highlights some of the potential mechanisms of SERCA2a dysfunction in HFpEF.

### mRNA and protein levels of SERCA2a in HFrEF

SERCA2a dysfunction has been well documented in the context of HFrEF [37], contributing to both systolic and diastolic dysfunction [11, 38, 39]. In their classic 1994 study, Hasenfuss et al. found a decrease in SERCA2a expression in failing human cardiac muscle tissue [40], which a 36% reduction in protein level of SERCA2a was noted in the failing muscle, compared to non-failing heart. Importantly, this study demonstrated that alterations in the force-frequency relationship of the myocardium was correlated with decreased SERCA2a levels.

Contrary to above findings, Schwinger et al. [41] interestingly found that protein levels of SERCA2a and PLN were unchanged in failing hearts due to dilated cardiomyopathy (DCM) compared with non-failing controls. Though the sample size for this study was relatively small (*n* = 9), they did observe a significant decrease in mRNA levels of SERCA2a, suggesting that the mechanisms underlying the dysregulation of SERCA2a and PLN in DCM hearts is at the transcriptional level as opposed to protein level, the findings agree with other studies [42–44]. It is worth to note, however, that reduced SERCA2a mRNA cannot directly explain reduced SERCA2a function. More recently, in the largest proteomics analysis of SERCA2a and PLN protein abundance to date [45], no significant difference was found in levels of SERCA2a and PLN in those with advanced HF due to DCM and ischaemic heart disease (IHD), compared to control hearts. These findings challenge the paradigm of reduced SERCA2a function in pre-clinical models and HF patients and gene therapy targeting it [46].

### Changes of SERCA2a in HFpEF

Disrupted Ca^2+^ cycling in HFpEF may arise from the reduced SERCA2a expression/activity, or due to altered regulation by PLN [35], for example, an increase in dephosphorylated PLN [47]. Moreover, evidence has indicated that SERCA2a can be activated independent of dissociation of the PLN/SERC2a complex [48, 49], suggesting that other mechanisms, such as post-translational modifications (PTMs) including acetylation [50], SUMOylation [51], phosphorylation [52], and S-glutathionylation [53]. Oxidative modifications include sulfonylation [54] and nitration [55] may contribute. PLN can undergo O-GlcNAcylation [56], which reduces PLN phosphorylation augmenting its inhibitory effect on SERCA2a, as well as S-nitrosylation [57] which can activate SERCA2a. Indeed, some of the studies in HFpEF models (Table 1) have reported oxidative PTMs to modulate SERCA2a function [58, 59]. Further investigation into the role of PTMs to (1) identify what is occurring specifically in the context of HFpEF would be beneficial, in particular as there is limited information on this, (2) whether it is aetiology-dependent and (3) what implications this has for any method to over-express SERCA2a ie gene therapy as PTM could reduce SERCA activity.

**Table 1:** Summary of the changes in activity and expression of SERCA2a in heart failure with preserved ejection fraction (HFpEF): preclinical and clinical evidence

Author, year	Type of study	Model/population	SERCA2a activity	SERCA2a expression	Post-translational modifications
Levitsky et al. 1993 [65]	Pre-clinical	Spontaneously hypertensive rats	Increased	Unchanged	
Okayama et al. 1997 [66]	Pre-clinical	Dahl salt sensitive rats	?	Decreased	
Belke et al. 2004 [82]	Pre-clinical	Db/db mice	?	NS decrease	NS decrease in PLN phosphorylation at Ser16 and increase in PLN
Wold et al. 2005 [83]	Pre-clinical	Sucrose-fed rats	Decreased	Unchanged	
Vasanji et al. 2006 [84]	Pre-clinical	Sucrose-fed rats	Decreased	Unchanged	Decreased PLN phosphorylation at Ser16 and Thr17
Li et al. 2006 [59]	Pre-clinical	Lep/Lep obese mice	Decreased	Unchanged	SERCA oxidation
Sakata et al. 2006 [67]	Pre-clinical	OLETF rats	?	Decreased	
Sakata et al. 2007 [69]	Pre-clinical	Aortic-banded rats	?	Decreased	
Sakata et al. 2007 [68]	Pre-clinical	OLETF rats	?	Decreased	
Lacombe et al. 2007 [70]	Pre-clinical	T1DM rats	?	Decreased	Reduced SERCA2a:PLN
Dupont et al. 2012 [71]	Pre-clinical	Spontaneously hypertensive rats	Decreased (NS)	Decreased	Reduction in PLN phosphorylation at Ser-16
Balderas-Villalobos et al. 2013 [58]	Pre-clinical	Sucrose-fed rats with MetS	Decreased	Unchanged	
Abdurrachim et al. 2014 [85]	Pre-clinical	HFD mice	?	Unchanged	Reduced PLN phosphorylation at Thr-17
Lima-Leopoldo et al. 2014 [86]	Pre-clinical	HFD mice	Unchanged	Unchanged	Reduced PLN phosphorylation at Ser16
Primessnig et al. 2016 [72]	Pre-clinical	Male Wistar rats	Initial decrease, followed by increase	Unchanged	Initial reduction in PLN phosphorylation, followed by increase
Curl et al. 2018 [73]	Pre-clinical	HHR	Increased	Decrease	Increased PLN phosphorylation at Thr17
Hohendanner et al. 2018 [74]	Pre-clinical	ZSF1 rats: lean (Ln) and obese (Ob)	NS difference	Decreased	
Rouhana et al. 2019 [75]	Pre-clinical	Rat model	Decreased	Unchanged	Increased PLN phosphorylation at Thr17 and increased PLN: SERCA2a
Miranda-Silva et al. 2020 [76]	Pre-clinical	Rat models (Zucker fatty spontaneously hypertensive) Hypertensive lean (control) Hypertensive obese	Decreased in obese rats (decreased SERCA: PLN)	Unchanged	Reduced PLN phosphorylation at Ser16
Kilfoil et al. 2020 [77]	Pre-clinical	Dahl salt-sensitive rats	Unchanged	Unchanged	
Frisk et al. 2021 [78]	Pre-clinical	Rat models (1) Diabetes and hypertension (2) Ischaemic (3) Hypertension	Reduced in (1) Increased in (2) Unchanged in (3)	Decreased in all	(1) No change in PLN (2) Increased PLN phosphorylation at Thr17 (3) Increased PLN phosphorylation at Ser16/Thr17
Smolgovsky et al. 2023 [47]	Pre-clinical	High fat diet + L-NAME mice	Decreased (decreased phosphorylated: total PLN)	Unchanged	Reduced PLN phosphorylation
Abudureyimu et al. 2023 [79]	Pre-clinical	High fat diet + L-NAME mice	Decreased	Decreased (increased PLN)	Reduced PLN phosphorylation
Abudureyimu et al. 2024 [80]	Pre-clinical	High fat diet + L-NAME mice	Decreased	Decreased (increased PLN)	Reduced PLN phosphorylation
Semmler et al. 2024 [81]	Pre-clinical	High fat diet + L-NAME mice	Increased	Unchanged	Increased PLN phosphorylation at Ser16. S-nitrosylation of PLN potential role
Stüdeli et al. 2006 [87]	Clinical	Heart transplant recipients with LVEF > 50%	?	Decreased (increased PLN expression)	
Lamberts et al. 2014 [88]	Clinical	Diabetic HFpEF myocardial tissue	Increased	Increased	Increased SERCA2a:PLN
Runte et al. 2017 [89]	Clinical	Myocardial LV biopsies from hypertensive heart disease (HHD) controls and HHD + HFpEF patients	?	Unchanged	
D’Assante et al. 2021 [63]	Clinical	Biopsies from control, chronic HFrEF and HFpEF patients	?	Unchanged—no difference between controls and HFpEF	

*?* SERCA2a activity unclear/not directly measured, *NS* not significant, *OLETF* Otsuka–Long–Evans Tokushima Fatty rat, *HHD* Hypertensive heart disease, *HHR* hypertrophic heart rats, *L-NAME* N(ω)-nitro-L-arginine methyl ester

Models of SERCA2a knockout (KO) and PLN mutation can provide valuable insights into how SERCA2a dysfunction can result in impair normal diastolic function. An approximately 35% decrease in SERCA2a expression and activity level in a heterozygous SERCA2a KO model led to a decreased maximal rate of relaxation [60]. Reduced Ca^2+^ re-uptake into the SR by SERCA2a impairs muscle relaxation through prolonging elevation of the cytosolic Ca^2+^, as well as diminishing the magnitude of the SR Ca^2+^ store available for the next cycle of excitation–contraction coupling, leading to a reduced CaT and impaired contractility. Excision of the gene encoding SERCA2a, *Atp2a2*, in mice resulted in severe diastolic dysfunction, as demonstrated through increases in several parameters including the following: LVEDP, the time constant of isovolumetric pressure decay (to 5 times its baseline value), and left atrial diameter [61]. Prolongation of CaT decay was also reported in transgenic mice expressing a missense PLN mutation, which trapped PKA and prevented phosphorylation of wild type PLN [62].

Although there are numerous literatures present documenting changes in SERCA2a expression and activity in HFrEF, there is significantly less information available for HFpEF [63]. This is due to multiple reasons, including inadequate animal models that do fully represent the clinical syndrome, as well as a lack of sample myocardial tissues [64]. However, in the limited studies available, there are opposing findings on changes, if any, in SERCA2a expression/activity and its correlation with HFpEF from both pre-clinical models [47, 58, 59, 65–86] and clinical studies [63, 87–89] (Table 1). Reasons for this disparity are not entirely clear; however, variations in HFpEF phenotypes, patient co-morbidities, disease progression, and study design and methodology are likely to be contributory factors.

Gene expression of proteins involved in regulating Ca^2+^ homeostasis was investigated in 31 cardiac allograft patients with a LVEF > 50% [87]. A significant inverse correlation was noted between SERCA2a gene expression and isovolumetric relaxation time (IVRT), suggesting the prolonged IVRT was due to reduced SERCA2a levels and thus reduced re-uptake into the SR. By contrast, cardiac tissue samples collected from non-diabetic patients with HFpEF undergoing coronary artery bypass grafting (CABG) showed an increase in the SERCA2a:PLN ratio, suggesting an increased rate of Ca^2+^ re-uptake into the SR and therefore a compensatory mechanism responsible [88]. Selby et al. also found, in non-diabetic patients with preserved EF, that there was a surprising increase in SR Ca^2+^ levels [90], suggesting that across different phenotypes of HFpEF (diabetic and non-diabetic), compensation may occur, though the exact compensatory mechanisms are unclear.

The changes leading to impaired Ca^2+^ homeostasis in HFpEF were investigated in rat models [78]. Three distinct groups of rats with HFpEF were used, each with a different aetiology driving the clinical syndrome, namely, hypertension, IHD and diabetes. It was found that SERCA2a levels were reduced in all three groups of rats, however reduced Ca^2+^ removal was only seen in diabetic rats. This finding is interesting for two reasons. Firstly, it highlights the importance of Ca^2+^ in contributing to diastolic dysfunction seen in HFpEF. Secondly, the variations in Ca^2+^ handling seen across these groups suggest differences in the level of SERCA2a function, thus highlighting the heterogeneity of HFpEF, with different underlying mechanisms giving rise to different clinical phenotypes [17] and even sub-phenotypes [91]. Intriguingly, in rats with IHD and hypertension, Ca^2+^ handling was compensated by increased PLN phosphorylation (which removes its inhibitory effect on SERCA2a). This was not seen in diabetic HFpEF rats and the reason why is poorly understood. Consistent with these results, one group reported elevated diastolic [Ca^2+^] and a slower CaT decline in db/db mice; however, they did not measure the protein expressions of SERCA2a and PLN [92]. It may indeed be that the chronic hyperglycaemia occurring in diabetes leads to O-GlcNAcylation of PLN, reducing its phosphorylation and therefore increasing inhibitory effects on SERCA2a [56]. Whilst hyperglycaemia has been identified as a contributing factor to left ventricular diastolic dysfunction, the extent of its impact is contingent upon both the degree and duration of elevated blood glucose levels [93]. Long-standing hyperglycaemia results in the production of advanced glycation end products, which cause an increase in collagen production, ultimately resulting in increased LV stiffness and reduced blood vessel compliance, both of which drive HFpEF [94]. In the acute setting, patients admitted with acute decompensated HFpEF who had non-diabetic hyperglycaemia (blood glucose ≥ 7.0 mmol/L) had increased risk of death from cardiac cause, as well as overall mortality [95]. Therefore, further study is required into the effects of long-standing hyperglycaemia on a molecular level, with a focus on the regulation of SERCA2a and PLN, in order to improve understanding of why these results were observed.

This observed effect suggests that changes, if any, in SERCA2a activity and expression in HFpEF patients are aetiology-dependent, for example a different change in Ca^2+^ cycling may be observed in a HFpEF patient with a hypertensive phenotype, compared with a diabetic patient. In the common scenario where there are multiple contributory aetiologies, often to the extent that the patient is classified under a cluster [22], matters become further complicated and determining the specific mechanisms by which Ca^2+^ levels are altered will be a challenging task. Considering this, further research into the underlying processes altering SERCA2a, whether direct or indirect, needs to be investigated in order to increase our understanding of aetiology-dependent changes in Ca^2+^ homeostasis in HFpEF.

Mitochondrial function plays a key role in cardiomyocyte Ca^2+^ homeostasis, with increased mitochondrial [Ca^2+^], increasing PLN activity, thereby inhibiting SERCA2a and raising the cytosolic [Ca^2+^] [96]. Two recent studies investigated the role of disrupted mitochondrial dynamics in the aetiology of HFpEF [79, 80]. HFpEF mice showed downregulation of SERCA2a and upregulation of PLN. F-box and leucine-rich repeat 4 (FBXL4), which is important in maintenance of mitochondrial function and mtDNA, improved SERCA2a function and reduced diastolic dysfunction in HFpEF, and further investigation is required into its role as a possible therapeutic target.

The reported cytosolic CaT in HFpEF models may provide valuable information regarding possible alterations in Ca^2+^ homeostasis. Multiple studies reported prolongation of the Ca^2+^ decay transient (reflective of decreased SERCA2a activity) [72, 97–99]; however, not all measured changes in protein expression of SERCA2a and PLN. A cardiorenal HFpEF model [72] found no change in SERCA2a protein content but progressive changes in SERCA2a activity, with initial reduced phosphorylation of PLN at 8 weeks, followed by increased phosphorylation at 24 weeks. Ca^2+^ decay was 77% slower in HFpEF rats at 24 weeks, and SR Ca^2+^ was reduced. Although the increased PLN phosphorylation at 24 weeks would be expected to enhance SERCA2a activity, there was no significant difference in the time constant of the CaT decay (reflective of SERCA2a activity) between HFpEF and control rats, suggesting that the prolonged CaT decay was not due to changes in SERCA2a activity. One possible explanation for this is that the impact of SERCA2a activity on SR Ca^2+^ content is not directly proportional. Specifically, a reduction in SERCA2a activity leads to a relatively smaller decrease in SR Ca^2+^ content than expected [100]. This reduced dependence means that whilst SERCA2a influences the amplitude of the systolic Ca^2+^ transient, its effect on SR Ca^2+^ content—and consequently on CaT decay—is less direct. Therefore, the prolonged CaT decay in HFpEF rats might be attributed to other factors, such as impaired SR Ca^2+^ handling or increased Ca^2+^ leakage (increased at 24 weeks in HFpEF rats), rather than changes in SERCA2a activity alone.

Kilfoil et al. [77] reported no change in SERCA2a expression or alterations in PLN phosphorylation at Ser-16 or Thr-17 in Dahl salt-Sensitive rats with HFpEF. Despite this, resting Ca^2+^ was increased and overall Ca^2+^ uptake was decreased, perhaps due to increased leak via phosphorylated RyR2 or increased activity of L-type Ca^2+^ channels. These findings underscore the complexity of Ca^2+^ handling in HFpEF and emphasise that, beyond SERCA2a, other mechanisms and proteins involved in Ca^2+^ regulation may be involved and require further investigation. Further evidence is needed to clarify the specific role of SERCA2a in HFpEF.

### Metabolic influences on SERCA2a function

Metabolic disturbances play a key role in HFpEF [101]. SERCA2a, which is the most energetically demanding enzyme in the cardiac contractile system, is particularly susceptible to any alterations in ATP metabolism [102]. In high-fat and high sugar (HFHS) mice, both at baseline and under conditions of increased cardiac workload, the absolute free energy of ATP hydrolysis (|ΔG∼ₚ|) was observed to be significantly lower than that required for normal SERCA2a function [103]. Although this study did not measure Ca^2+^ dynamics, previous work [104] has reported a prolongation in the rate of cytosolic Ca^2+^ decline in rats treated with glycolysis inhibitor iodoacetamide (11 ms longer compared with controls) in order to decrease (|ΔG∼ₚ|). The evidence above, combined with the fact that SERCA2a is highly ATP-dependent for its role in re-uptake of Ca^2+^ into the SR, suggest that diminished ATP availability directly compromises its function.

Building on these findings, further investigation into Ca^2+^ transport dynamics in cardiomyocytes subjected to metabolic stress is warranted. Understanding the degree to which CaT and cytosolic Ca^2+^ are altered could provide insights into the contribution of SERCA2a dysfunction under conditions of ATP depletion. Moreover, evaluation of the effects of decreased ATP levels, in the context of metabolic HFpEF, on other calcium-handling proteins, such as RyR2 and NCX, could help delineate whether the defects in Ca^2+^ handling are primarily driven by SERCA2a impairment or involve broader dysregulation across multiple Ca^2+^-handling pathways.

Computational modelling may offer a powerful tool to simulate the complex interactions between metabolic dysregulation, SERCA2a function and calcium handling in HFpEF. One model revealed Ca^2+^ dynamics to be the primary factor influencing LV contractile behaviour [105]. The group used previously reported parameters of LV function in obese ZSF1 rats formed the primary data to build this model and included measurements of: EF, PeakP (peak pressure), maxdP (max. rate of pressure rise), and tau (time constant of isovolumic relaxation). Such models could incorporate parameters for ATP levels, calcium fluxes, and protein kinetics, providing a detailed mechanistic understanding of how metabolic stress impacts cardiac function. This in silico approach could also help identify potential therapeutic targets aimed at preserving or restoring SERCA2a function and overall calcium homeostasis in the context of HFpEF.

### Inflammation and SERCA2a

Inflammation is a key driver in the pathogenesis of HF and to a much greater extent in HFpEF, in comparison to HFrEF [106]. It is possible that inflammatory cytokines are responsible for the over-production of growth factors promoting myocardial fibrosis in HFpEF [107], with two of the most common pro-inflammatory cytokines [108, 109], TNF-*α* and IL-6, being implicated in the downregulation of SERCA2a and diastolic dysfunction [109]. It must be emphasised that this group utilised HL-1 cells, which do not fully represent adult cardiomyocytes, and there was no measurement of changes in the expression of other Ca^2+^ handling proteins.

Putko et al. reported a notable rise in serum TNF-*α* and TNFR1 levels in HFpEF patients [110]. Furthermore, TNFR2 levels, compared to both healthy controls and HFrEF patients, were also significantly higher in HFpEF patients. Elevated TNFR1 levels have been associated with a greater risk of incidence of HFpEF than HFrEF, whilst no significant association was observed between TNFR1 levels and HFrEF [111], highlighting the increased importance of TNF-driven inflammation in patients with a preserved EF.

Multiple studies have reported that TNF-*α* could downregulate SERCA2a and affect normal cardiomyocyte Ca^2+^ homeostasis [112–115]. One possible underlying mechanism responsible for the impact of TNF-*α* on SERCA2a is decreased expression of *atp2a2*, the gene encoding SERCA2a, due to inhibition of the gene promoter through binding of a transcription factor [115]. Nuclear factor kappa-light-chain-enhancer of activated B cells (NF-*κ*B) is predominantly found in the cytoplasm which is naturally inhibited by IκB [116]. Treatment with TNF-α resulted in a significant increase in the phosphorylation of IκB kinase (IκK) and subsequent degradation of IκB in cardiomyocytes. These findings indicated TNF-α induced activation of the IκK/IκB/NF-κB signalling cascade, ultimately resulting in the nuclear translocation of NF-*κ*B and reduced SERCA2a gene promoter activity [115].

Other findings suggest TNF-*α* causes an increase in DNA methyltransferase, increasing methylation of the SERCA2a gene promoter, leading to reduced SERCA2a expression [112]. It would, therefore, be reasonable to propose that one of the main ways in which TNF-*α* affects SERCA2a is through transcriptional regulation, and thus treatments targeting these signalling pathways, for example by preventing excess methylation [112], may be useful.

The presence of HFpEF has been identified as a strong predictor of increased IL-6 levels and patients with HFpEF are more likely to have IL-6 levels on the top end of the spectrum [117]. IL-6 was shown to cause a significant decrease in SERCA gene expression in treated neonatal rat ventricular myocytes [118, 119]. Interestingly, IL-6 was also found to reduce SERCA2a activity through reduced PLN phosphorylation at Thr-17 residue [120]. In acute decompensated HFpEF, elevated IL-6 was associated with increased death due to cardiovascular and non-cardiovascular causes [121], indicating IL-6 is a potential therapeutic target as well as a prognostic marker in HFpEF patients.

High-fat diet (HFD) in mice induces diabetes and systemic inflammation, followed by the production of pro-inflammatory cardiac macrophages, which release interleukin-1β (IL-1β) [122]. IL-1β, similar to IL-6, impairs cardiac diastolic function through exerting negative effects on SERCA2a [123] and was shown to reduce PLN expression at both transcript and protein level [124]. A direct decrease in SERCA2a gene expression in cultured cardiomyocytes treated with IL-1β [125] was also reported, and injection of IL-1β in mice resulted in increased LVEDP and IVRT—both indicative of diastolic dysfunction. A positive correlation was identified between levels of NOD-like receptor pyrin domain-containing protein 3 (NLRP3) inflammasome and the degree of diastolic dysfunction in HFpEF. NLRP3 causes activation of caspase-1 which leads to production of IL-1β [126]. Increased IL-1β leads to oxidative stress and the production of mitochondrial reactive oxygen species (ROS), both of which negatively impact SERCA2a. It is therefore possible that antagonism of IL-1β may be beneficial in HFpEF patients through restoration of SERCA2a function.

Mixed results have been observed in trials investigating the effects of administering anakinra, an IL-1 antagonist. Interestingly, patients with HFpEF responded differently to anakinra treatment depending on their resting LVEF [127]. Those with a LVEF ≥ 60% showed greater improvement in cardiorespiratory fitness. Further investigation is required to investigate the underlying mechanisms of this varied response, as well as animal and human studies of the effects of IL-1 blockade on SERCA2a/PLN activity and expression in HFpEF patients.

IL-17, produced by T-lymphocytes, leads to the production of TNF, IL-6 and IL-1β, all implicated in inflammatory pathways in HFpEF and the downregulation of SERCA2a activity and expression. IL-17D, a cardiac subtype of IL-17, was found increased in HFpEF patients [128]. Importantly, IL-17 supresses SERCA2a through the activation of NF-*κ*B [129].

### Oxidative stress and SERCA2a

Increased ROS production and/or diminished antioxidant production can lead to oxidative stress [130]. Increased ROS is involved in the development of HFpEF [131]. Biopsies of cardiac muscle tissues from HFpEF patients have shown signs of increased ROS and oxidative stress [132, 133]. It has been suggested that ROS can have a direct impact on cardiac diastolic function through modification of microRNA that leads to inhibition of SERCA2a gene transcription [134]. Excess superoxide reacts with nitric oxide (NO) resulting in the production of peroxynitrite (ONOO^−^) which can damage DNA, increase IL-6 receptor expression and reduce the activity of SERCA2a [135]. Additionally, peroxynitrite leads to the activation of protein phosphatase 2a (PP-2a), which reduces phosphorylation of PLN at the Ser-16 residue, reducing SERCA2a activity [136].

Oxidative stress is a major contributor to metabolic syndrome (MetS), which is closely associated with HFpEF and can affect the function of SERCA2a. Older [58] and more recent [137] studies have reported decreased SERCA2a activity due to oxidative stress in rats with MetS. ROS play a central role in the development of other associated co-morbidities in HFpEF patients, including ageing [138], diabetes [139], and hypertension [140]. Renal proximal tubule cells in mice with angiotensin II-induced hypertension exhibited heightened superoxide production, resulting in SERCA2 dysfunction through mechanisms affecting the cysteine 674 residue. This SERCA2 dysfunction is believed to induce endoplasmic reticulum stress that results in increased activity of Na^+^/K^+^-ATPase and thus increased water retention, contributing to hypertension. This underscores the importance of recognising HFpEF as a systemic syndrome. Merely addressing SERCA2a to restore cardiac Ca^2+^ is, alone, insufficient treatment. Additional factors, such as hypertension induced by oxidative stress affecting SERCA2 in other locations, for example the kidneys, necessitate a wider range of targeted therapies, for which further research is required.

Nicotinamide adenine dinucleotide phosphate oxidases, also known as NOXs, are enzyme complexes found in almost all tissue cells and in neutrophils [141]. NOXs are involved in redox reactions that lead to the production of ROS. One particular isoform of interest is myocardial NOX2, which, despite being involved in normal physiology of the heart, can contribute to the progression of disease conditions such as arrhythmias, coronary artery disease, hypertension and HF [141, 142]. Using a cardiomyocyte-specific NOX2 transgenic mouse model, our study showed that overexpression of NOX2 leads to increase the activity of SERCA2a through inhibition of protein-phosphatase 1 (PP-1), which phosphorylates PLN thus preventing its inhibition of SERCA2a [143]. Given that its expression levels are raised in myocardium of HFpEF patients [144], NOX2 could be a potential therapeutic target.

Dysfunction of the arterial wall endothelium is driven by ROS and therefore by NOX2, which generates ROS. We investigated the effect of endothelial dysfunction on cardiac remodelling in mice with NOX2 overexpression specific in endothelium [145]. A significant isolated decrease in LV diastolic function was noted, partially due to NOX2-induced inflammation leading to endothelial cells adopting a mesenchymal phenotype, which can drive myocardial fibrosis [146]. The varying effects of NOX2 on the heart shown in these two studies highlight their complexity, warranting the need for further investigation about the cell-type specific contribution of NOX2, ultimately with the aim of finding therapies to reduce inflammation and cardiac remodelling leading to HFpEF.

### Changes of SERCA2a in HFpEF vs. HFrEF: what’s different, why and what’s next?

Unlike the relatively consistent picture of altered SERCA2a activity in human (and pre-clinical models) of HFrEF, in HFpEF the relation between SERCA2a and abnormal cardiomyocyte Ca^2+^ handling is less straightforward. Based on the evidence outlined above, it is speculated that SERCA2a function may vary among heterogeneous HFpEF human subgroups and among different pre-clinical animal HFpEF models. The discrepancies shown imply that simply quantifying SERCA2a expression may not suffice to elucidate its role in specific HFpEF phenotypes. To address this, we propose that future studies should focus and improve on several key areas to enhance our understanding of the role of SERCA2a in HFpEF: (a) inclusion of diverse HFpEF phenotypes and the further advancement of animal models to reflect these; (b) longitudinal studies that monitor changes in SERCA2a function over time in relation to clinical presentation, disease progression and therapeutic intervention, in order to determine correlation between SERCA2a function and severity of HFpEF, though direct measurement of SERCA2a activity and expression via invasive endomyocardial biopsy is practically difficult in the context of a longitudinal study; (c) the effect of post-translational modifications on SERCA2a; (d) *in* *silico* models of HFpEF to examine changes in SERCA2a and Ca^2+^ cycling; and (e) interventional studies to evaluate how modification of SERCA2a activity through drug or gene therapy affects outcomes in HFpEF. However, it will be difficult to standardise these studies if SERCA2a dysfunction is aetiology/clinical course-dependent.

## SERCA2a as a therapeutic target

Drug therapy and gene therapy represent two fundamentally different approaches and thus will be discussed separately. All of the current and complete drug and gene therapy trials targeting SERCA2a in HF are summarised in Tables 2 and 3, respectively.

**Table 2:** Drugs targeting SERCA2a in HFrEF and HFpEF

Therapy	Molecular target/mechanism	Trial dose	Clinical trial phase	Outcomes	National clinical trial (NCT) number	References
Istaroxime	Dual: 1. Increased inotropy through suppression of the Na + /K + -ATPase pump 2. Stimulation of SERCA2a leading to increased lusitropy	0.5 μg/kg/min	Early phase 1 trial investigating use in HFpEF patients	No effect on myocardial relaxation—suggestion of higher doses?	NCT02772068	[148, 153]
		0.5–1.5 μg/kg/min, dependent on safety	Phase 2 trial investigating safety and efficacy in acute HFrEF	Improved PCWP and possible improvement in diastolic function	NCT00838253	[154]
		0.5–1.5 μg/kg/min	HORIZON-HF: phase 2 trial in HFrEF patients	Improvement in diastolic function	NCT00616161	[151]
		1.0 μg/kg/min	Phase 2 trial investigating safety and efficacy in acute HFrEF	Improvements seen in blood pressure and on echocardiogram	NCT04325035	[194]
		0.5 or 1.0 μg/kg/min	Phase 2 trial investigating safety and efficacy in acute HF	Improved systolic and diastolic cardiac function with minimal adverse effects	NCT02617446	[195]
BMS-986231	Cardiomyocyte nitroxyl donor Activates SERCA through post-translational modifications (glutathiolation) of cysteine residues	Dose escalation: 3.0, 5.0, 7.0 and 12.0 μg/kg/min	Phase 2a investigating use in hospitalised HFrEF patients	Positive inotropy and lusitropy	NCT02157506	[196, 197]

**Table 3:** Gene therapies targeting SERCA2a via percutaneous intra-coronary administration

Vector	Viral titration	Controls	Clinical trial phase	Outcome	National clinical trial (NCT) number	Reference
AAV1-SERCA2a	3.0 × 10^13^ vg fixed dose	–	MUSIC-HFpEF: phase 1b pilot in HFpEF patients	Ongoing– est. primary completion 08/25	NCT06061549	[168]
AAV1-CMV-SERCA2a	1.0 × 10^13^ DRP single dose	Placebo	AGENT-HF phase 2 trial in HFrEF patients	No improvement noted. Trial terminated early due to lack of benefit demonstrated in CUPID-2 trial	NCT01966887	[161]
AAV1/SERCA2a	Serial dose escalation (DRP) Very low: 1.0 × 10^11^ Low: 6.0 × 10^11^ Mid: 3.0 × 10^12^ High: 1.0 × 10^13^	Placebo (NaCl)	Phase 1/2 trial in HFrEF patients	No safety concerns and possible benefit supporting further trials	NCT00454818	[162]
AAV1/SERCA2a	3.0 × 10^13^ or 4.5 × 10^13^ single dose	SRD-001 matching placebo	MUSIC-HFrEF1: phase 1/2: in HFrEF patients	Ongoing—est. primary completion 12/25	NCT04703842	[142]
AAV1/SERCA2a	1.0 × 10^13^ DRP single dose	Placebo	CUPID-2: phase 2b trial in HFrEF patients	No significant improvement in disease outcome	NCT01643330	[160]
AAV1/SERCA2a	2.5 × 10^13^ DRP single dose	Placebo (NaCl)	Phase 1–2 for advanced HFrEF	Terminated early—no results	NCT02346422	[198]
AAV1/SERCA2a	1.0 × 10^13^ DRP single dose	Placebo (NaCl)	Phase 2 trial in patients with LVAD	Early termination	NCT00534703	[163]
AAV1/SERCA2a	Low dose/high dose	No intervention—control	MUSIC-DMD Phase 1	Not yet recruiting	NCT06224660	[199]

*DRP* DNase-resistant particle, *vg* viral genome, “*–*”no control group

### Istaroxime, PST3093 and compound 8

Identified for its stimulatory effect on SERC2a in 2005 [147], istaroxime is a derivative of androstenedione [148] and has been investigated in both HFrEF and HFpEF. Istaroxime operates via a dual mechanism: inhibition of the Na^+^/K^+^-ATPase (NKA) leading to increased inotropy and activation of SERCA2a, which improves lusitropy [149]. The activation of SERCA2a by istaroxime is contingent on the presence of PLN and occurs independently of secondary messenger signalling processes via cAMP/PKA. Istaroxime interacts directly with the PLN/SERCA2a complex, resulting in dissociation of PLN from SERCA2a, increasing Ca^2+^ re-uptake into the SR [150]. Shah et al. investigated the effect of istaroxime on diastolic stiffness in patients with acute HF, reporting a significant decrease in end-diastolic elastance [151]. Torre et al. found that, in diabetic rat models, through activation of SERCA2a, istaroxime improves Ca^2+^ homeostasis and so increases myocardial relaxation [152]. Unfortunately, Sarma et al., in their recent study (NCT02772068, the only clinical trial investigating istaroxime in humans with HFpEF), found that istaroxime had no significant effect on myocardial relaxation and ventricular filling pressures [153]. They did however report a rise in systolic blood pressure, suggesting istaroxime caused a moderate improvement of cardiac contractility. Perhaps the dual mechanism of istaroxime diminishes its lusitropic effect due to preferential action on NKA and may provide an explanation for the increased inotropic effect reported by this group.

Another key issue with istaroxime is its very short half-life, which is less than 1 h [154]. PST3093 is a metabolite of istaroxime and poses several major benefits. It is entirely selective for SERCA2a [155], with no action on the Na^+^/K^+^-ATPase pump, reducing the likelihood of off-target interactions and toxicity. Furthermore, it has a half-life of approximately 9 h [156] and has already been shown to improve cardiac function in rats with diabetic cardiomyopathy [156].

Recently, the effects of a PST3093 derivative named “compound 8” were investigated in the setting of reduced SERCA2a expression and diastolic dysfunction, induced by streptozotocin (STZ) administration in rats. The results showed that both IV infusion (in acute decompensated HF) and oral administration (in chronic HF) of compound 8 resulted in an improvement in diastolic function and overall cardiac hemodynamics [157], with the absence of any significant off-target effects. Notwithstanding, knowledge of the precise function and effects of PST3093 and compound 8 on SERCA2a in HFpEF are very limited and require further study. Considering the recent failure of istaroxime to improve cardiac relaxation, further investigation into its metabolites and derivatives, with careful translation to clinical trials, may introduce a new avenue for modulating diastolic function in HFpEF.

### Gene therapy targeting SERCA2a

Gene therapy using adeno-associated virus (AAV) vector increases SERCA2a function and so increases the reuptake of Ca^2+^ into the SR [158]. Multiple studies showed administration of AAV/SERCA2a in pre-clinical HFpEF animal models result in an improvement in cardiac function [67–69, 159]. However, human gene therapy trials targeting SERCA2a have only been conducted in HFrEF patients and have, on the whole, been unsuccessful [160, 161]. The phase 1/2 CUPID 1 trial [162] demonstrated improvements in three key parameters in HFrEF patients: symptom, function and biomarker, as well as a satisfactory safety profile. This success led to the initiation of the larger, multinational CUPID 2 trial [160]. Unfortunately, CUPID 2 did not replicate the positive outcomes observed in CUPID 1 and was unsuccessful in improving disease outcomes in HFrEF patients. Based on the results from CUPID 2, two ongoing trials were terminated early [161, 163].

Several factors have been hypothesised for the failure of AAV1/SERCA2a gene therapy in CUPID 2 and understanding these issues will provide critical insights for improving future gene therapy approaches, in particular those targeting HFpEF. Firstly, there was a discrepancy between the proportion of empty viral capsids (those without the single-stranded DNA) in CUPID 1 and 2, at 85% and 25% respectively [160]. These empty capsids may serve as a decoy and reduce immune system interference [164]. The presence of low-level neutralising antibodies or other interfering substances in the body can reduce the effectiveness of gene therapy. Using fewer empty capsids necessitates even higher doses to achieve the same amount of gene transfer, which can be challenging and may be partially responsible for the failure seen in CUPID 2. Secondly, the presence of neutralising antibodies can reduce the effectiveness of gene therapy. Development of new vectors that can evade the immune system and demonstrate superior targeting to cardiac tissues is necessary [165]. Thirdly, current delivery methods may not effectively transport the gene to all necessary areas of the myocardium, limiting treatment efficacy. By addressing the identified challenges—improving delivery efficiency, optimising the use of empty capsids, and developing immunoevasive vectors—gene therapy could potentially be more successful in upregulating SERCA2a and restoring Ca^2+^ re-sequestration into the SR in HFpEF.

There have been several reports of BNP reducing SERCA2a function. BNP activates protein kinase G (PKG) and thus raises levels of cGMP, which inhibits the calcineurin-NFAT signalling pathway that increases SERCA2a activity [166]. Zhai et al. noted decreased activity and expression of both endogenous and exogenous SERCA2a following administration of BNP in an animal model [167]. In contrast to CUPID 1, patients enrolled in CUPID 2 were required to have elevated BNP/NT pro-BNP—given that BNP may affect exogenous SERCA2a; it is possible that this was a contributing factor to the failure of the trial. BNP levels are generally lower in HFpEF patients, suggesting this population may reap greater benefit from SERCA2a gene therapy. In the past, there have been no SERCA2a gene therapy trials in HFpEF and the phase I MUSIC-HFpEF trial, currently recruiting, is particularly significant as it is the first gene therapy trial assessing the impact of enhanced SERCA2a activity in HFpEF patients [168]. Results from this study are eagerly awaited and will demonstrate the effects, if any, of increased SERCA2a in the myocardium on cardiac function, as well as informing future SERCA2a gene therapy endeavours for HFpEF.

Viral uptake, expressed in copies of vector per μg, is approximately ~ 20,000–350,000 in pre-clinical animal models [169], compared to < 20–561 in CUPID and < 10–192 in CUPID 2, for which the maximum doses administered were 3 × 10^12^ DRP and 1 × 10^13^ DRP respectively. In comparison, six weeks following administration of 1 × 10^13^ DRP of AAV2/1SERC2a in sheep, uptake reached 2649 ± 1225 copies per mg DNA [170]. These data suggest that gene transduction in animal models is not predictive of human myocardial transduction efficiency and indeed the authors of CUPID 2 recognised that higher doses may yield greater benefit. Therefore, dosage optimisation to improve gene transduction is a critical focus for advancing gene therapy trials.

In contrast to previous gene therapy trials targeting SERCA2a, the data that contributed to the MUSIC-HFpEF trial were based on novel bioengineering technologies that created a mini-heart HFpEF model that is human-specific [171]. HFpEF was induced in human ventricular cardiac tissue strips (hvCTS), to measure contractility and stiffness, and a human ventricle-like organoid chamber (hvCOC). The hvCOC aims to replicate key physiological features of the human ventricle and allows measurement of parameters of ventricular pump function [172]. In the human mini-heart model, viral titre was a key factor determining SERCA2a expression after transduction with AAV1-SERCA [171], underscoring the importance of careful dose optimisation to maximise transduction efficiency. The fixed dose of 3 × 10^13^ vg per patient being used in the MUSIC-HFpEF trial was influenced by this group’s work in three key ways, (1) prior knowledge that the dose of 1 × 10^13^ used in CUPID 2 was ineffective to improve outcomes, (2) translation from mini-heart findings: 1 × 10^14^ vg per cell to maintain structural integrity and efficient transduction, and (3) scaling to the human heart with approximately 2–3 billion cardiomyocytes [171, 173].

Enhanced SERCA2a activity can influence the CaT in two main ways. Firstly, whilst increased SERCA2a activity increases the SR Ca^2+^ content and therefore increases the amplitude of the CaT, this also shortens the duration of the CaT. Since contraction follows the rise in intracellular [Ca^2+^], a shorter CaT might reduce the amplitude of contraction [174]. Thus, beyond a certain point, further stimulation of SERCA2a may not enhance contractility. Additionally, excessive SERCA2a overexpression could reduce the amplitude of the CaT by overly buffering cytoplasmic Ca^2+^ [175], highlighting the complexity of targeting SERCA2a activity in HF therapy.

A specific safety concern regarding the use of gene therapy is the possible proarrhythmogenic effect of the increased inotropy caused by upregulation of SERCA2a, which may cause excessive Ca^2+^ uptake into the SR, triggering spontaneous leakage of Ca^2+^ into the cytosol via RyR2 [176] leading to delayed after depolarisations (DADs) and subsequent arrhythmias. This effect, however, was not observed in numerous animal studies [177–182] with overexpression of SERCA2a. A possible mechanism responsible for this was suggested by Sato et al., who used a computational model of a rabbit ventricular myocyte to investigate the effects of altered SERCA function on Ca^2+^ cycling [183]. They found that a small increase in SERCA function led to more frequent and intense Ca^2+^ release from the SR, which may act as an arrhythmogenic substrate. However, when SERCA function was increased to a higher level, anti-arrhythmic effects were observed. This is due to the fact that the increased [Ca^2+^]_SR_ means increased opening of RyR2 and stronger Ca^2+^ release flux, which in turn increases the rate of Ca^2+^ uptake from the cytosol and can prevent spread of Ca^2+^ released from one calcium release unit (CRU; specialised regions of the SR containing clusters of RyR2) to adjacent CRUs to trigger further release events. Considering that there was an observed Ca^2+^ mishandling in HFpEF in the presence of preserved of T-tubules [78], modulation of SERCA2a may be crucial in stabilising intracellular Ca^2+^ dynamics in HFpEF and preventing arrhythmias.

## HFpEF: what does the future hold?

### SGLT2 inhibitors

The current literature on the effects of SGLT2i on SERCA2a is limited. One study reported that empagliflozin increased SERCA2a protein levels in STZ-induced DM rats [184]. Another study noted an increase in PLN phosphorylation, as well as an increased SERCA2a: PLN, in a T2DM induced mouse model [185]. Benefits seen in these mice included an improvement in diastolic function as well as positive anti-diabetic effects. Similarly, recent findings suggest that in ZSF1-obese rats, empagliflozin treatment enhances PKA-mediated phosphorylation of PLN at Ser-16, thereby relieving the inhibitory effect on SERCA2a and increasing SR Ca^2+^ re-uptake [186], with no change in SERCA2a expression reported.

SGLT2i may also target inflammatory pathways in HFpEF, with a recent meta-analysis noting a decrease in many inflammatory markers, such as TNF-*α*, IL-6, and CRP in animal models following administration of SGTLT2i [187]. These may indirectly increase SERCA2a function. Empagliflozin given in an animal model of HFpEF led to reduced NLRP3 activation [188], which in turn leads to a reduction in IL-1β levels and so increases SERCA2a function. Furthermore, treatment of empagliflozin in T2DM patients at high risk of cardiovascular disease led to a reduction in IL-1β secretion due to reduced NLRP3 inflammasome activation [189]. Whilst this supports the idea that SGLT2i may indirectly have a positive effect on SERCA2a, similar trials will be required in HFpEF patients, as the results of the aforementioned studies may not necessarily apply to this population.

### GLP-1 agonists

A recent study (STEP-HFpEF) found that in non-diabetic patients with obesity-related HFpEF, treatment with the GLP-1 agonist, semaglutide, resulted in significant improvements across multiple parameters [7, 190]. CRP was also significantly reduced, suggesting semaglutide exerts anti-inflammatory effects [7]. Considering the key role inflammatory processes play in HFpEF, and in downregulation of SERCA2a, the action of GLP-1 agonists in reducing inflammation in HFpEF may be beneficial in progressing treatments for the syndrome.

Interestingly, GLP-1 could reduce cardiac diastolic dysfunction through increasing SERCA2a activity and expression, as well reducing phosphorylation of the ryanodine receptor, which increases diastolic SR Ca^2+^ uptake [191]. One study found GLP-1 agonists improved cardiac function through targeting smooth muscle cell SERCA in the coronary vasculature [192]. Furthermore, treatment with the GLP-1 agonist Exendin-4, increased PLN phosphorylation and thus SERCA2a activity, ameliorating the negative effect of high glucose on reducing SERCA2a mRNA and protein levels in cultured cardiomyocytes [193]. Whilst this may seem promising, the presence of MetS appears to reduce the extent to which GLP-1 agonists increase SERCA activity [192]. Given that HFpEF patients often have co-morbidities such as T2DM, the true effectiveness of GLP-1 agonists remains to be seen, and further investigation into the mechanisms underlying the diminished effect on SERCA is required.

## The changing approach to the HFpEF patients

The complexity of mechanisms underlying the clinical syndrome is reflected across the wide spectrum and heterogeneity of HFpEF patients. Indeed, disrupted cardiomyocyte Ca^2+^ homeostasis due to SERCA2a dysfunction may be important in contributing to the diastolic dysfunction seen in HFpEF. However, solely aiming to restore cardiomyocyte Ca^2+^ cycling through targeted therapies aimed at SERCA2a will likely be inadequate in reducing mortality and treating HFpEF effectively. For example, the direct and indirect pathways leading to SERCA2a dysfunction, as well as factors independent of SERCA2a, all contribute to altered Ca^2+^ sequestration and diastolic dysfunction in the failing heart with a preserved EF. Achieving effective treatment for HFpEF may require a more holistic and personalised management which comprises a “cocktail” of drugs strategically selected to mitigate inflammation, target co-morbidities such as diabetes and hypertension, and modulate SERCA2a activity and expression. Such an approach would acknowledge the complexity of HFpEF and therefore be essential in developing treatment algorithms containing targeted therapies with the ultimate aim of reducing mortality.

## Conclusions

Whilst the existing literature provides a basic foundation for exploring the potential involvement of SERCA2a dysfunction in HFpEF, most data point to associations, not direct evidence, linking SERCA2a with diastolic dysfunction. The evidence remains very limited and often conflicting, which features the significant gaps in our understanding of the exact role of SERCA2a in HFpEF. Despite preliminary findings suggesting that some degree of SERCA2a dysfunction could contribute to HFpEF, the underlying mechanisms and extent of its involvement remain unclear. This review highlights the need for well-designed pre-clinical studies and robust clinical trials to further investigate the potential role of SERCA2a dysfunction in HFpEF. Whilst various drugs have been shown to improve SERCA2a activity and calcium sequestration in the failing heart, these are often in animal models and their direct relevance to human cases remains unknown. The outcomes of current clinical trials targeting SERCA2a in HFpEF are awaited.

## Data Availability

No datasets were generated or analysed during the current study.

## Abbreviations

AKAP: A-kinase anchoring protein
cAMP: 3′,5′-Cyclic adenosine monophosphate
CaT: Calcium transient
DRP: DNase resistant particle
GLP-1: Glucagon-like peptide 1
HFmrEF: Heart failure with mildly reduced ejection fraction
HFpEF: Heart failure with preserved ejection fraction
HFrEF: Heart failure with reduced ejection fraction
IL-1: Interleukin-1
IL-6: Interleukin-6
NLRP3: NOD-like receptor pyrin domain-containing protein 3
NOX: Nicotinamide adenine dinucleotide phosphate (NADPH) oxidase
PLN: Phospholamban
ROS: Reactive oxygen species
SGLT2i: Sodium-glucose co-transporter protein 2 inhibitors
SERCA2a: SarcoEndoplasmic Reticulum Ca^2+^-ATPase
TNF-α: Tumour necrosis factor-α
